# COVID-19 in Hospitalised Children and Adolescents: Review of the First Pandemic Year at Vilnius University Hospital Santaros Klinikos

**DOI:** 10.15388/Amed.2021.29.1.8

**Published:** 2022-07-26

**Authors:** Inga Ivaškevičienė, Kamilė Donielaitė-Anisė, Virginija Žilinskaitė, Daiva Vaičiūnienė, Rimvydas Ivaškevičius

**Affiliations:** Clinic of Children’s Diseases, Institute of Clinical Medicine, Faculty of Medicine, Vilnius University, Vilnius, Lithuania; Vilnius University Hospital Santaros Klinikos, Pediatric Center, Santariškių str. 4, Vilnius LT-08406, Lithuania; Clinic of Children’s Diseases, Institute of Clinical Medicine, Faculty of Medicine, Vilnius University, Vilnius, Lithuania; Vilnius University Hospital Santaros Klinikos, Pediatric Center, Santariškių str. 4, Vilnius LT-08406, Lithuania; Clinic of Children’s Diseases, Institute of Clinical Medicine, Faculty of Medicine, Vilnius University, Vilnius, Lithuania; Pediatric Emergency, Intensive care and Anaesthesiology Center, Santariškių str. 7, Vilnius LT-08406, Lithuania; Clinic of Children’s Diseases, Institute of Clinical Medicine, Faculty of Medicine, Vilnius University, Vilnius, Lithuania; Pediatric Emergency, Intensive care and Anaesthesiology Center, Santariškių str. 7, Vilnius LT-08406, Lithuania; Clinic of Children’s Diseases, Institute of Clinical Medicine, Faculty of Medicine, Vilnius University, Vilnius, Lithuania; Vilnius University Hospital Santaros Klinikos, Pediatric Center, Santariškių str. 4, Vilnius LT-08406, Lithuania

**Keywords:** COVID-19, children, adolescents, hospitalisation

## Abstract

**Background.:**

Since the start of the pandemic with SARS-CoV-2 virus, very little data was known about clinical features and outcomes of COVID-19 in children and adolescents not only in Lithuania, but also in other European countries. This study was started in collaboration with 82 participating healthcare institutions across 25 European countries, using a well-established research network—the Paediatric Tuberculosis Network European Trials Group (ptbnet). This multinational, multicentre cohort study was performed during the first wave of the pandemic, between April 1 and April 24, 2020. Each participating country was allowed to continue further research individually encompassing brighter time limits and using the same methodology. We present here data of children hospitalised at Vilnius University Hospital Santaros Klinikos (VUH SK) during the first year of the pandemic.

**Materials and methods.:**

We included all paediatric patients with PCR confirmed SARS-CoV-2 infection who were hospitalised at VUH SK. The study was performed between March 12, 2020 and March 12, 2021. A standardised data collection spreadsheet was used to record epidemiological, clinical and treatment data.

**Results.:**

A total of 104 patients were included in the study. The median age of participants was 5 years (IQR 1.0-11.0, range 0-17 years). Males accounted for 50 (48%) of all patients. The average duration of hospitalisation was 3 days. Ten (9.6%) patients had pre-existing medical conditions. Among all hospitalised patients 16 (15%) were asymptomatic, 5 (4.8%) were treated in intensive care unit (ICU). The most common symptoms among COVID-19 patients were pyrexia 71 (68%) followed by upper respiratory tract infection 49 (47%) and gastrointestinal symptoms 33 (32%). Among the entire cohort only 3 (3%) patients required oxygen support, but none of them was started on continuous positive airway pressure (CPAP), mechanical ventilation or extracorporeal membrane oxygenation (ECMO). None of the patients admitted to ICU needed inotropic support. There was no fatal outcome.

**Conclusions.:**

Our data indicate that COVID-19 may affect children of any age. The COVID-19 disease was usually mild in hospitalized children and adolescents. The most common clinical findings of COVID-19 were pyrexia and symptoms of upper respiratory tract infection. Severe COVID-19 disease cases when oxygen support or treatment in ICU was required were very rare. No patient received antiviral drugs for Covid-19 treatment. There was no fatal outcome due to COVID-19 in our study population.

## Santrauka.

**Įvadas.:**

Nuo pat SARS-CoV-2 pandemijos pradžios Lietuvoje, kaip ir kitose Europos šalyse, labai stigo duomenų apie klinikinę COVID-19 ligos išraišką ir jos baigtis vaikams. Pristatomas tyrimas buvo inicijuotas Vaikų tuberkuliozės tinklo (ptbnet) Europos tyrimų grupės ir atliktas bendradarbiaujant su kitais 82 medicinos centrais iš 25 skirtingų Europos valstybių. Šis tarptautinis multicentrinis tyrimas buvo vykdomas per pirmą pandemijos bangą, nuo 2020 metų balandžio 1 iki 24 dienos. Kiekvienas tyrime dalyvavęs centras turėjo teisę tęsti tyrimą pagal tą pačią metodologiją, pasirinkti individualų tyrimo laikotarpį. Straipsnyje pristatome duomenis apie COVID-19 liga sirgusius vaikus, kurie buvo hospitalizuoti Vilniaus universiteto ligoninės Santaros klinikose pirmaisiais pandemijos metais.

**Medžiaga ir metodai.:**

Į tyrimą buvo įtraukti visi vaikai, kuriems PGR metodu patvirtinta SARS-CoV-2 infekcija ir jie buvo hospitalizuoti Vilniaus universiteto ligoninės Santaros klinikose. Tyrimas vykdytas vienerius metus – nuo 2020 m. kovo 12 d. iki 2021 m. kovo 12 d. Naudojant elektorinę duomenų anketą buvo renkami duomenys apie epidemiologinius, klinikinius ir vaikų gydymo ypatumus.

**Rezultatai.:**

Iš viso į tyrimą buvo įtraukti 104 vaikai. Vidutinis vaikų amžius buvo 5 m. (IQR 1,0–11,0, ribos 0–17 m.). Iš visų dalyvių berniukų buvo 50 (48 %). Vidutinė hospitalizacijos trukmė – 3 dienos. Dešimt (9,6 %) pacientų sirgo lėtinėmis ligomis. Iš visų hospitalizuotų pacientų 16 (15 %) buvo besimptomiai, 5 (4,8 %) buvo gydyti intensyvios terapijos skyriuje. Dažniausiai pasitaikę klinikiniai COVID-19 simptomai buvo karščiavimas 71 (68 %), viršutinių kvėpavimo takų infekcijos 49 (47 %) bei virškinimo trakto pažeidimo požymiai 33 (32 %). Iš visų tiriamųjų 3 (3 %) pacientams reikėjo deguonies terapijos, tačiau nė vienam iš jų neprireikė nuolatinio teigiamo slėgio (CPAP) terapijos, dirbtinės plaučių ventiliacijos ar ekstrakorporinės oksigenacijos (ECMO). Nė vienam intensyvios terapijos skyriuje gydytam pacientui nereikėjo vazoaktyvių vaistų. Nebuvo registruota letalių baigčių.

**Išvados.:**

Mūsų duomenys rodo, kad COVID-19 liga gali sirgti įvairaus amžiaus vaikai. COVID-19 eiga hospitalizuotų vaikų dažniausiai buvo nesunki. Dažnai pasitaikę klinikiniai COVID-19 simptomai buvo karščiavimas ir viršutinių kvėpavimo takų infekcijos požymiai. Sunki COVID-19 eiga, kai reikėjo deguonies terapijos ar gydymo intensyvios terapijos skyriuje, pasitaikė labai retai. Nė vienam pacientui nebuvo skirta antivirusinių vaistų. Tyrimo kohortoje nebuvo registruota letalių baigčių.

## Introduction

In December 2019 a novel beta-coronavirus (SARS-CoV-2), causing unusual pneumonia, was identified in Wuhan, China. The disease was later termed COVID-19 and spread rapidly across the globe. The first cases in Europe were identified in late January 2020. Soon after this, COVID-19 pandemic was declared by the World Health Organisation (WHO) [1].

Despite the fact that the numbers of COVID-19 cases were constantly growing worldwide, data on children and adolescence were scarce at the beginning of the pandemic. There was data from China showing that children are less severely affected as compared to adults, but most of the published data had small sample sizes, data regarding the management and clinical course of COVID-19 in children were lacking [2-4]. Similarly, publications from Europe and North America were focusing on the adult population, therefore very little information was known about children and adolescents [5, 6].

In order to get more data on COVID-19 in European children a multinational multicentre study was set up, using a well-established research network—the Paediatric Tuberculosis Network European Trials Group (ptbnet). This study was the first one, which gave key data on COVID-19 in children in Europe [7]. In total 582 children with PCR confirmed SARS-CoV-2 infection were included in the final analysis across 25 European countries. The data showed that COVID-19 is generally a mild disease in children, however some children may develop severe disease. Out of all patients 8% required treatment in ICU, fatal outcomes were overall rare. This study was conducted for one month only, as the main aim was to rapidly capture key data on COVID-19 in children in Europe, to aid physicians in Europe and in other geographical locations with service planning and allocation of resources.

Vilnius University Hospital Santaros Klinikos being the partner of the initial study contributed to the final results, however less than 10 patients were included, as this was the number of hospitalised patients sick with COVID-19 during the study time period in the Vilnius region. Since the start of the pandemic in March 2020 Lithuania applied strict rules of infection control, therefore the COVID-19 morbidity rate, especially in a paediatric population, was very low. In total there were 26 children diagnosed positive with SARS-CoV-2 during the first month of the pandemic in the whole country [8]. In order to get more data on COVID-19 in children hospitalised at VUH SK, it was essential to continue the study. To our knowledge, this is the first study that provides a detailed overview of COVID-19 in hospitalised children in Lithuania. The aim of our study was to overview the epidemiological, clinical and treatment data of children hospitalised in VUH SK due to confirmed COVID-19 during the first year of the pandemic.

## Materials and methods

This study is a continuation of the above-mentioned European multicentre cohort study. The original European study was performed for one month only, but each participating centre was allowed to continue further research individually, choosing their own time limits. Our study was conducted during the first year of the pandemic and covered the period between March 12, 2020 and March 12, 2021. The study was approved by the Vilnius Regional Committee of Biomedical Research (Approval No.2020/4-N1-1225-704).

All paediatric patients aged 18 years or younger positive for SARS-CoV-2 infection (proven by PCR) and hospitalised in VUH SK were included in the study. A standardised data collection spreadsheet was used to record epidemiological, clinical and treatment data.

Diagnosis of upper respiratory tract infection was based on clinical signs and symptoms, encompassing any of the following: coryza, pharyngitis, tonsillitis, otitis media, or sinusitis. Lower respiratory tract infection was based on clinical signs and auscultation findings. Gastrointestinal symptoms included diarrhoea and/or vomiting. Pyrexia was defined as a body temperature of at least 38.0°C.

### Statistical analysis

Statistical analyses were performed using MS Excel and IBM SPSS Statistics software version 25.0. In children younger than 1 year, age was calculated as a fraction of a whole year; from 1 year of age, age was rounded to the nearest year. The normality of data distribution was assessed with the Shapiro–Wilk test. The clinical endpoint was the need for admission to an intensive care unit. Odds ratios were calculated with 95% confidence intervals. Significance was accepted with a p value < 0.05.

## Results

A total of 104 patients with positive SARS-CoV-2 infection were included in the study. All COVID-19 cases were confirmed by PCR. The median age of the study individuals was 5 years (IQR 1.0-11.0, range 0-17 years). Age was nonnormally distributed (W=0.6629, p<0.0001). Males accounted for 50 (48%) of all participants (table 1).

**Table 1.** Baseline characteristics in the entire cohort and by the requirement of the ICU admission

	Entire cohort (n=104)	Not admitted to ICU (n=99)	Admitted to ICU (n=5)	Odds ratio (95% CI)	Relative risk	p-value
Male	50 (48%)	49 (49%)	1 (20%)	ref.		
Female	54 (52%)	50 (51%)	4 (80%)	3.92 (0.42-36.33)	3.7	0.2291
**Age groups**						
<1 yr.	31 (30%)	30 (30%)	1 (20%)	0.58 (0.06-5.36)	0.59	0.6271
1-4 yrs.	18 (17%)	18 (18%)	0	-	-	-
5-10 yrs.	18 (17%)	17 (17%)	1 (20%)	1.26 (0.13-12.03)	1.25	0.8381
11-18 yrs.	37 (36%)	34 (34%)	3 (60%)	2.87 (0.46-18.00)	2.72	0.2609
**Symptoms**						
Pyrexia	71 (68%)	68 (69%)	3 (60%)	0.68 (0.11-4.30)	0.87	0.6854
Headache	6 (6%)	5 (5%)	1 (20%)	4.7 (0.44-50.22)	3.96	0.2004
Muscle pain	15 (14%)	15 (15%)	0	-	-	-
Gastrointestinal	33 (32%)	33 (33%)	1 (20%)	0.5 (0.05-4.65)	0.6	0.5425
Upper respiratory tract infection	49 (47%)	49 (49%)	0	-	-	-
Lower respiratory tract infection	11 (11%)	10 (10%)	3 (60%)	13.35 (1.99-89.70)	5.94	0.0077
Asymptomatic	16 (15%)	16 (16%)	0	-	-	-
**Chest X-ray**	14 (13%)	10 (10%)	4 (80%)	35.6 (3.62-350.41)	7.92	0.0022
Suggestive of pneumonia	5 (36%)	3 (30%)	2 (50%)	2.33 (0.22-25.25)	1.67	0.4856
Oxygen support	3 (3%)	1 (1%)	2 (40%)	65.33 (4.56-935.17)	39.6	0.0021

A parent as a source of infection was identified in 43 (41.3%) of cases. Other sources, usually individuals outside the family (e.g., schoolmates, teachers or friends), were found in 53 (51%) cases. In 8 (7.7%) the source of infection was unknown.

The average duration of hospitalisation was 3 days. There was no statistically significant difference found between the patient’s age and the duration of hospitalisation.

Ten (9.6%) patients had preexisting medical conditions such as chronic pulmonary disease (1), neurological disorders (2), malignancy (3), congenital heart disease (1), chronic kidney disease (1) and systemic rheumatic disease (2). Three of them were receiving immunosuppressive medication at the time of COVID-19 diagnosis. Just one patient with the chronic neurologic disease was admitted to ICU due to worsening pulmonary function, he didn’t receive immunosuppressive therapy. There was no statistically significant difference between the duration of hospitalisation among patients with preexisting medical conditions as compared to previously healthy individuals.

Among all hospitalised patients 16 (15%) were asymptomatic and were hospitalised due to other reasons. Five (4.8%) individuals experienced severe COVID-19 and were treated in ICU.

The most common symptoms among COVID-19 patients were: pyrexia in 71 (68%), followed by upper respiratory tract infection in 49 (47%) and gastrointestinal symptoms in 33 (32%). Symptoms of lower respiratory tract infection were more common among patients admitted to ICU, this result was statistically significant (p <0.007).

Chest X-ray was done in 14 (13%) of cases. Of those, 5 (36%) had changes consistent with pneumonia. None had changes suggestive of acute respiratory distress syndrome (ARDS). Among the entire cohort only 3 (3%) patients required oxygen support, but none of them was started on continuous positive airway pressure (CPAP), mechanical ventilation or extracorporeal membrane oxygenation (ECMO). None of the patients admitted to ICU needed inotropic support. There was no fatal outcome.

No patient received chloroquine, hydroxychloroquine, remdesivir, lopinavir/ritonavir, zanamivir, or ribavirin. No steroids or IVIG were prescribed for COVID-19 treatment.

## Discussion and conclusions

To our knowledge, this is the first study in Lithuania that provides a comprehensive overview of COVID-19 in hospitalised children and adolescents. This study was continued for one year, it covered the first and second waves of the pandemic.

One of the major goals of the study was to analyse what is the burden of COVID-19 in hospitalised paediatric patients. As this is a hospital-based study, it doesn’t represent the whole paediatric population, especially those children who are less sick and do not seek medical attention, but it allows us to evaluate what was the picture of the severe course of COVID-19 in children.

Our data show that COVID-19 may affect children of any age, starting from newborns to adolescents. The vast majority of children (3 (60%)) admitted to ICU were children of 11-18 years old. This suggests that adolescents may be more severely ill. Nevertheless, we have to keep in mind that these numbers are very small, therefore such a conclusion can’t be made.

Despite the fact that our data were collected at a hospital setting, we found that 15 (16%) of cases were asymptomatic. It means that patients were admitted to the hospital due to other reasons, but were found to be positive for SARS-CoV-2. Data in the literature are diverse: some publications provide very similar results, showing that asymptomatic infection was found in 16%, others give higher rates, reaching up to 30% [7, 9-11]. A recent publication from the US shows completely different results, where the prevalence of asymptomatic infection varied from 0% to 2.2% [12]. The authors conclude that asymptomatic paediatric prevalence was significantly associated with the weekly incidence of COVID-19 in the general population. This could explain different findings in various studies.

Pyrexia (51.2%) and signs of upper respiratory tract infection (47%) were the most commonly reported symptoms in our patients. Interestingly, one-third of the entire cohort reported having gastrointestinal symptoms such as diarrhoea or vomiting. Similar results are provided in many other publications where fever and cough are predominant clinical features [9-11, 13]. Data on gastrointestinal symptoms varies (from 13% to 79%), but many authors recognise these symptoms as an important part of paediatric COVID-19 [7, 14, 15].

Symptoms of lower respiratory tract infection were more common in children admitted to ICU, showing that these patients are more severely ill. Overall, only 5 (4.8%) children were treated in ICU, and despite the fact that 2 patients required oxygen support, none of them needed CPAP, mechanical ventilation or ECMO. This is a very important finding showing that in the vast majority of cases COVID-19 was a mild disease in children and adolescents. Another important issue is oxygen therapy: out of the entire cohort only 3 children required additional oxygen support. This is a very low number of patients as compared to adults. It is a very important finding for best practice-oriented service planning, meaning that paediatric COVID-19 patients may require intensive care or additional oxygen support, but not in large numbers.

The most comforting finding was that there was no fatal outcome in our study population. To our knowledge, there is no case of COVID-19 related paediatric death registered in the whole country for the entire period of the pandemic [8]. Unfortunately, other studies show that rarely children may die due to severe SARS-CoV-2 infection, but overall mortality rates are low, especially as compared to the adult population [16-18]. One of the recent publications analysed child COVID-19 mortality in seven different countries (USA, United Kingdom, Italy, Germany, Spain, France and South Korea). Data showed that deaths from COVID-19 in children were rare, accounting 0.17 cases per 100 000 population [16]. Data from the USA indicates that a history of comorbidity, supersedes age, gender, and race/ethnicity is a risk factor for in-hospital paediatric COVID-19 death [19]. In our study, out of all patients with underlying medical conditions, just one was admitted to ICU and later recovered. Having just one case we can neither support, nor contradict data from the USA.

There were no critically ill patients in our study population, therefore there was no need to try treatments with different combinations of antiviral drugs. Nevertheless, there are uncertainties regarding drug treatment options for COVID-19 in many European countries and further paediatric studies are needed [7].

This study was started soon after the pandemic was declared when physicians were experiencing unusual workloads and a lot of uncertainty. Therefore, the initial idea was to collect the most basic data on paediatric COVID-19, which would be less time-consuming. A small number of variables collected is the main limitation of our study. It would be very important to evaluate if other respiratory viruses were present, to collect more precise clinical and laboratory data. The other limitation is the small number of severe cases of COVID-19, therefore it’s not possible to make reliable conclusions. It would be important to keep collecting data for a longer time period or to conduct the study in several centres of Lithuania. Further research is also needed as the SARS-CoV-2 mutates and new strains become dominant.

Nevertheless, this study is the first one in the country, which gives a review of the COVID-19 course in hospitalised paediatric patients.

In conclusion, our data indicate that COVID-19 may affect children of any age. The COVID-19 disease was usually mild in hospitalised children and adolescents. The most common clinical findings of COVID-19 were pyrexia and symptoms of upper respiratory tract infection. Severe COVID-19 disease cases when oxygen support or treatment in ICU was required were very rare. No patient received antiviral drugs for COVID-19 treatment. There was no fatal outcome due to COVID-19 in our study population.
